# DICER-ARGONAUTE2 Complex in Continuous Fluorogenic Assays of RNA Interference Enzymes

**DOI:** 10.1371/journal.pone.0120614

**Published:** 2015-03-20

**Authors:** Mark A. Bernard, Leyu Wang, Souvenir D. Tachado

**Affiliations:** 1 Department of Target Biology, Pfizer Oligonucleotide Therapeutics Unit, Cambridge South Campus, Cambridge, Massachusetts, United States of America; 2 Department of Protein Biology, Pfizer Global Biotherapeutics Technology Unit, Cambridge North Campus, Cambridge, Massachusetts, United States of America; 3 Department of Medicine, Division of Pulmonary, Critical Care and Sleep Medicine, Beth Israel Deaconess Medical Center and Harvard Medical School, Boston, Massachusetts, United States of America; CNRS UMR7622 & University Paris 6 Pierre-et-Marie-Curie, FRANCE

## Abstract

Mechanistic studies of RNA processing in the RNA-Induced Silencing Complex (RISC) have been hindered by lack of methods for continuous monitoring of enzymatic activity. “Quencherless” fluorogenic substrates of RNAi enzymes enable continuous monitoring of enzymatic reactions for detailed kinetics studies. Recombinant RISC enzymes cleave the fluorogenic substrates targeting human thymidylate synthase (*TYMS*) and hypoxia-inducible factor 1-α subunit (*HIF1A*). Using fluorogenic dsRNA DICER substrates and fluorogenic siRNA, DICER+ARGONAUTE2 mixtures exhibit synergistic enzymatic activity relative to either enzyme alone, and addition of TRBP does not enhance the apparent activity. Titration of AGO2 and DICER in enzyme assays suggests that AGO2 and DICER form a functional high-affinity complex in equimolar ratio. DICER and DICER+AGO2 exhibit Michaelis-Menten kinetics with DICER substrates. However, AGO2 cannot process the fluorogenic siRNA without DICER enzyme, suggesting that AGO2 cannot self-load siRNA into its active site. The DICER+AGO2 combination processes the fluorogenic siRNA substrate (*K*_m_=74 nM) with substrate inhibition kinetics (*K*_i_=105 nM), demonstrating experimentally that siRNA binds two different sites that affect Dicing and AGO2-loading reactions in RISC. This result suggests that siRNA (product of DICER) bound in the active site of DICER may undergo direct transfer (as AGO2 substrate) to the active site of AGO2 in the DICER+AGO2 complex. Competitive substrate assays indicate that DICER+AGO2 cleavage of fluorogenic siRNA is specific, since unlabeled siRNA and DICER substrates serve as competing substrates that cause a concentration-dependent decrease in fluorescent rates. Competitive substrate assays of a series of DICER substrates *in vitro* were correlated with cell-based assays of *HIF1A* mRNA knockdown (log-log slope=0.29), suggesting that improved DICER substrate designs with 10-fold greater processing by the DICER+AGO2 complex can provide a strong (~2800-fold) improvement in potency for mRNA knockdown. This study lays the foundation of a systematic biochemical approach to optimize nucleic acid-based therapeutics for Dicing and ARGONAUTE2-loading for improving efficacy.

## Introduction

RNA interference brings about silencing of specific genes by sequence-specific degradation of mRNA [1]. Micro RNA precursors are derived from primary RNA transcripts that can form intramolecular hairpins (pri-miRNA), which can be cleaved in the nucleus by the endonuclease DROSHA to produce hairpin loops with a 3’ dinucleotide overhang called pre-microRNAs [2]. Pre-miRNAs are then exported via nuclear export receptor, Exportin-5 into the cytoplasm [2,3] for endonucleolytic processing by the RNA-Induced Silencing Complex (RISC) consisting of DICER, ARGONAUTE and the human immunodeficiency virus-1 transactivating response RNA-binding protein TRBP [4]. The cytoplasmic endonuclease, DICER binds the 3’-overhang and cleaves in a staggered fashion to yield an additional 3’ dinucleotide overhang on the opposite end in which each strand of the resulting double-stranded short interfering RNA (siRNA) is 21- to 23-nt long [5,6]. A complex of DICER and the dsRNA-binding protein TRBP is reported to be involved in microRNA processing and AGO-loading [7], although TRBP is dispensable *in vitro* [8,9]. The endonuclease ARGONAUTE2 (AGO2) binds one of the strands as the Guide Strand and is thus programmed to make single-strand cleavages in mRNA at a site complementary to the Guide Strand residing in the active site of AGO2 [10].

In the current enzyme kinetics study, we describe novel enzymatic assays of DICER and AGO2-loading with continuous monitoring of fluorescence intensity. New mechanistic insights were gained using fluorogenic dsRNA substrate molecules. This study provides a systematic biochemical approach to optimize nucleic acid-based therapeutics for Dicing and AGO2-loading in order to improve efficacy. Using purified recombinant human RNAi proteins to reconstitute the RISC complex *in vitro*, DICER loads the endonuclease ARGONAUTE2 with a double-stranded siRNA possibly in a direct transfer mechanism, resulting in enzymatic cleavage. Competitive enzyme kinetic assays of AGO2 loading were correlated with mRNA knockdown in cell-based assays targeting hypoxia inducible factor 1-α (HIF1A). This report describes a new technology for continuous assay of DICER and AGO2 enzymatic activities that enables biochemical evaluation of new designs of nucleic-acid therapeutic candidates. The RNA interference apparatus of cells may allow new therapies: nucleic-acid based drugs that target previously undruggable targets by specifically cleaving the messenger RNA.

## Materials and Methods

Ribonuclease H (*Escherichia coli*) was from Promega (Madison, WI). BODIPY FL-dextran conjugate was from Invitrogen (Carlsbad, CA). NBS-coated 384-well plates with black wells were from Corning Life Sciences (Lowell, MA). Mineral oil (Nujol oil) was from PerkinElmer Life And Analytical Sciences, Inc. (Waltham, MA). iCycler iQ 96-well PCR plates, Microseal ‘B’ Film and an iCycler iQ5 multicolor real-time PCR instrument were from Bio-Rad Laboratories (Hercules, CA). SeeBlue Plus2 pre-stained standard was from Invitrogen (Carlsbad, CA). Single-stranded oligonucleotide designs are shown in Table 1. RNA oligonucleotides with the 5’-phosphate modification were synthesized and purified by Invitrogen (Carlsbad, CA). RNA oligos BoGD664AS, BoPD664S, BoPD664s-dAdG and BoPsi664S were individually conjugated to BODIPY FL dye with an aminohexyl linkage to the 5’-phosphate and purified to >97% full-length product by Invitrogen (Carlsbad, CA). Oligoribonucleotide S955 was synthesized and purified by Dharmacon (Lafayette, CO). AllStars Negative Control siRNA (a non-silencing siRNA) was from Qiagen, Inc. (Valencia, CA). Unlabeled DICER substrates were synthesized and purified by IDT (Coralville, IA) or by the Pfizer Oligonucleotide Therapeutics Unit (Cambridge, MA). The hepatocarcinoma cell line Huh-7.5 was from the American Type Culture Collection (Manassas, VA).

**Table 1:** Oligoribonucleotide sequences of fluorogenic substrates.

ORN	Strand	Sequence
BoGD664AS	AS	BODIPY FL-aminohexyl-5'-PO_4_-CUGGUGAGGAAUGGGUUCACAAAUCAG**tt**-3'
GD664S	S	5'-PO_4_-CUGAUUUGUGAACCCAUUCCUCACCAG-3'
GD664dAdG-S	S	5'-PO_4_-CUGAUUUGUGAACCCAUUCCUCACC**ag**-3'
BoPD664S	S	BODIPY FL-aminohexyl-5'-PO_4_-CUGAUUUGUGAACCCAUUCCUCACCAG-3'
BoPD664S-dAdG	S	BODIPY FL-aminohexyl-5'-PO_4_-CUGAUUUGUGAACCCAUUCCUCACC**ag**-3'
PD664AS	AS	5'-PO_4_-CUGGUGAGGAAUGGGUUCACAAAUCAG**tt**-3'
BoPsi664S	S	BODIPY FL-aminohexyl-5'-PO_4_-CUGAUUUGUGAACCCAUUC**tt**-3'
Psi664AS	AS	5'-PO_4_-GAAUGGGUUCACAAAUCAG**tt**-3'
S955	S	5’-GGAGCUUGAAGGAUAUUGUCAGUCUUUAGGGGUUGGGCUGGAUGCCGAGG-3’
Bo995Ra	AS	BODIPY FL-aminohexyl-5’-PO_4_-CUGACAAUAUCCUUCAAGCUCCUU-3'
Bo995Rb	AS	BODIPY FL-aminohexyl-5’-PO_4_-CUAAAGACUGACAAUAUCCUUCAAGCUCCUU-3'
Bo995Rb5	AS	BODIPY FL-aminohexyl-5’-PO_4_-CUAAAGACUG-3'

2’-Deoxyribonucleotides are shown in boldface underscored lower case.

S: Sense strand.

AS: Anti-sense strand.

BoGD664 (Guide-strand labeled dsRNA): GD664S * BoGD664AS duplex.

BoGD664dAdG (Guide-strand labeled dsRNA): GD664dAdG-S * BoGD664AS duplex BoPD664 (Passenger-strand labeled dsRNA): BoPD664S * PD664AS duplex.

BoPD664dAdG (Passenger-strand labeled dsRNA): BoPD664dAdG-S * PD664AS duplex BoPsi664 (Passenger-strand labeled siRNA): BoPsi664S * Psi664AS duplex.

All data are contained in the paper. Baculovirus expressing human AGO2 enzyme, DICER enzyme and TRBP used in this study had been discarded by Pfizer, Inc. during the company’s reorganization. All other materials are commercially available. Other researchers can reproduce the current study using similar materials. DICER is available from Life Technologies (catalog no. K3600–01 and K3650–01) and Genlantis (San Diego, CA; catalog no. T510001). AGO2 is available from Sino Biological Inc. (catalog no. 11079-H07B-10). TRBP (TARBP2) is available from OriGene Technologies (catalog no. PH301043) and Abcam (catalog no. ab181920).

### Melting analysis of dsRNA strands (fluorescence vs. UV absorbance detection) and fluorescence quenching upon duplex formation

Duplexes (10 μM in TE Buffer) were denatured at 95°C in PCR tubes on VWR Modular Heating Blocks, and the blocks were transferred to the bench top for gradual annealing. Duplexes were diluted to 200 nM in Mg-Containing Buffer (50 mM HEPES, pH 7.5, 20 mM KCl, 1 mM MgCl_2_) or “Divalent Cation-Free Buffer” (50 mM HEPES, pH 7.5, 20 mM KCl, 5 mM EDTA). Similarly, dilutions of fluorescent ssRNA strand (BoPsi644S) were prepared for linear calibration of fluorescence intensity as a function of temperature vs. concentration of unquenched ssRNA strand. Fluorescence intensity was monitored using a 7900HT Fast Real-Time PCR System (Applied Biosystems, Foster City, CA). The instrument was configured for Absolute Quantitation mode to monitor channel #7 during thermal denaturation and annealing; temperature was slowly adjusted (25–4°C, 4–95°C and 95–4°C) in Standard Block mode (ramp rate of 1%, *i*.*e*. -0.59°C/min for cooling and +0.50°C/min for warming). Absorbance (λ = 260 nm) was also monitored for duplexes undergoing thermal denaturation and annealing in 1-cm quartz mini-cells using a Cary 100 Bio UV-visible spectrophotometer configured with the 6×6 Multicell Block Peltier Series II and Temperature Controller accessory (Agilent Technologies, Santa Clara, CA); temperature was adjusted slowly from 20–4°C, 4–90°C and 90–4°C at a ramp rate of 0.50°C/min.

### Protein expression and purification

Recombinant human RISC proteins (AGO2, DICER1 and the TARBP2 variant of TRBP) were expressed in an insect baculovirus system using the Titerless Infected cells Preservation and Scale-up (TIPS) method [11–13]. Baculoviruses were generated using the Bac-to-Bac system (Invitrogen). Human AGO2, DICER and TRBP were engineered to express the full-length protein with a 6×histidine tag (N-terminal tag for AGO2 and TRBP, C-terminal tag for DICER), and TEV protease cutting site was inserted between the ORF and His tag for purification purposes. All constructs were verified with double strand sequencing.

RISC proteins were purified using three common steps: nickel affinity, reverse nickel affinity and size-exclusion chromatography. Briefly, 1L of cells was suspended in 100 mL lysis buffer (Buffer NA) containing 50 mM Tris pH 8.0, 500 mM NaCl, 5% glycerol, 5 mM Tris(2-carboxyethyl)phosphine (TCEP) and protease inhibitors. The cells were lysed with a microfluidizer (Watts Fluidair). The insoluble protein and cell debris were sedimented through a 2-hour centrifugation at 40,000×*g* (4°C). The supernatant was filtered and loaded with 5 mM imidazole onto a HisTrap HP column (GE Bioscience) equilibrated with 0.5% Buffer NB (Buffer NA containing 1M imidazole). For AGO2 purification, both buffer NA and NB also contained 0.1% Triton X-100. The column was step-washed/eluted with 0.5, 2.5, 5, 10, 30% buffer NB. The fractions containing corresponding proteins were pooled. TEV protease was added to a final concentration 50 units/mg protein and dialyzed against Buffer NA overnight (4°C) to remove the His tag. The dialyzed proteins were then applied to a HisTrap HP column, and the unbound proteins were pooled and concentrated to 3 mL. The concentrated proteins were further purified through a size-exclusion column Superdex 200 16/60 equilibrated with gel filtration buffer (50 mM Tris pH 8.0, 300 mM NaCl, 0.5 mM EDTA, 10% glycerol and 2 mM DTT) with a flow-rate at 1 mL/min. Fractions containing corresponding proteins were pooled and concentrated. The protein concentrations were measured using Bio-Rad Bradford assay with BSA as standard. Proteins were flash-frozen in liquid nitrogen and stored at -80°C. Purified proteins were analyzed on a NuPAGE Bis-Tris 4–12% gel with MES running buffer followed by Coomassie Blue staining (Invitrogen, Carlsbad, CA).

### Continuous enzymatic assay of DICER activity and DICER-dependent AGO2-loading activity

BODIPY FL-dextran was diluted in Assay Buffer (50 mM HEPES, pH 7.5, 10 mM KCl, 2.0 mM MgCl_2_, 0.5 mM EDTA, 0.5 mM EGTA, 1 mM phenylmethylsulfonyl fluoride and 1 mM dithiothreitol), to several concentrations ranging from 0–350 nM of fluorescent dye. BODIPY FL-dextran was applied to a 384-well plate (40 μL/well), and mineral oil (20 μL/well) was layered on top of the aqueous solution to prevent evaporation. Fluorogenic substrates were diluted in Assay Buffer, applied to a 384-well plate, and mineral oil (20 μL/well) was layered on top of the solution. Assays that include EDTA controls used Assay Buffer containing the indicated concentration of EDTA, 20 mM KCl or NaCl and no MgCl_2_. Well contents were de-gassed by centrifuging the plate for 60 s. under vacuum using the swinging-bucket A-2-VC rotor of a Vacufuge Plus centrifuge (Eppendorf, Westbury, NY). Substrate equilibration was monitored (excitation 488 nm, emission 520 nm, cut-off 515 nm, high gain) in SpectraMax Gemini or M5 spectrofluorometers (Molecular Devices, Sunnyvale, CA) for 2 hr at 37°C. Recombinant human RNAi protein mixtures (AGO2, DICER, TRBP or combinations thereof) in Assay Buffer or *E*. *coli* RNAse H were applied to wells and mixed using a multi-channel pipette. The final reaction mixtures were de-gassed by centrifuging the plate again for 60 s. under vacuum in the A-2-VC rotor of an Eppendorf Vacufuge Plus centrifuge, and fluorescence intensity was monitored in a microplate spectrofluorometer for 18 hrs at 37°C.

### Thermal denaturation analysis of reaction products

For each digestion, 25 μL of the aqueous contents of duplicate reaction wells were transferred to a 96-well PCR plate and sealed with Microseal ‘B’ Film. The plate was centrifuged for 2 min at 4°C (1000×*g* in a GH-3.8 rotor of an Allegra 6KR centrifuge; Beckman-Coulter, Palo Alto, CA). The plate was placed in an iCycler iQ5 Real Time system (Bio-Rad Laboratories, Hercules, CA) and brought to 37°C followed by slow annealing (-0.5°C/min) to 4°C. The plate was slowly warmed from 4–22°C (1°C/min) followed by 22–24°C (0.25°C/min). Thermal denaturation (ramped slowly from 24–95°C at 1°C/min) was recorded using the FITC filter set. The temperature dependence of the fluorescence intensity was taken into account: at each temperature the raw fluorescence intensities of the digestion products were converted to the concentration of unquenched BODIPY FL dye by computing the linear relationship between fluorescence intensity vs. BODIPY FL-ssRNA concentration at each respective temperature.

### Enzymatic AGO-loading in the DICER-AGO2 complex by competitive substrate assay

The fluorogenic AGO substrate BoPsi664 in Assay Buffer was applied (20 μL/well) to a 384-well plate, and mineral oil (20 μL/well) was layered on top of the solution. Well contents were de-gassed by centrifuging the plate for 60 s. under vacuum using the swinging-bucket A-2-VC rotor of a Vacufuge Plus centrifuge (Eppendorf, Westbury, NY). The plate was incubated 2hr at 37°C. Unlabeled competing substrate in Assay Buffer was injected into the lower phase and mixed (15 μL/well). The plate was degassed as before, and substrate equilibration was monitored (excitation 488 nm, emission 520 nm, cut-off 515 nm, high gain) using a SpectraMax Gemini spectrofluorometer (Molecular Devices, Sunnyvale, CA) for 2 hr at 37°C. Recombinant human DICER+AGO2 in Assay Buffer was injected (5 μL/well) and the lower phase was mixed using a multi-channel pipette. The final reaction mixtures containing DICER+AGO2 (30 nM each), the fluorogenic siRNA BoPsi664 (80 nM) and indicated concentration of unlabeled DICER substrate or unlabeled siRNA in Assay Buffer were de-gassed as before, and fluorescence intensity was monitored in the microplate spectrofluorometer for 18 hr at 37°C. The apparent initial rates of increased fluorescence intensity were plotted as a function of the logarithm of competing substrate concentration. *IC*_50_ values were calculated using GraphPad Prism software (GraphPad Software, San Diego, CA).

### Cell assays for knockdown of *HIF1A*

Hepatocarcinoma cell line Huh-7.5 was treated with DICER substrates (Table 1) or non-silencing AllStars Negative Control siRNA. Cell extracts were analyzed for knockdown of *HIF1A* mRNA using the Panomics branched DNA (bDNA) assay (Affymetrix, Santa Clara, CA) according to the manufacturer’s instructions. The *EC*_50_ values for mRNA knockdown were calculated using GraphPad Prism software (GraphPad Software, San Diego, CA).

## Results

### Principle of fluorogenic assays for DICER and AGO2 enzymatic activity

**Fig 1:**
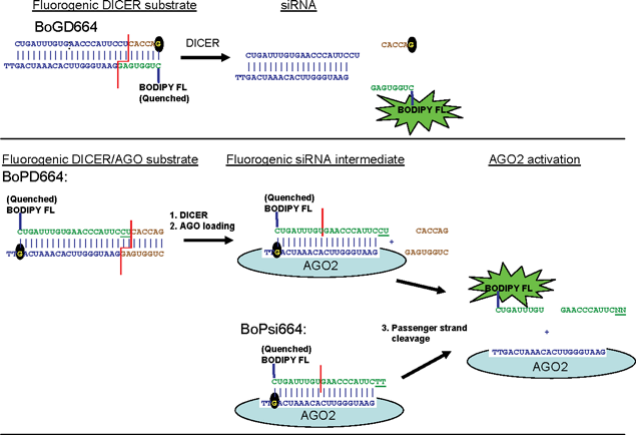
Principle of the fluorogenic enzyme assays. Fluorescent dye (BODIPY FL) conjugated to RNA duplexed to anti-sense oligonucleotides is quenched by the guanine base of the guanosine nucleotide (black background) on the strand directly opposite the fluorescent dye. Enzymatic cleavage of RNA strand produces a thermodynamically unstable duplex product with low melting temperature, which dissociates at assay temperature (37°C) resulting in increased fluorescence intensity.

The new fluorogenic assay allows continuous monitoring for enzyme kinetics studies. The fluorescent dye BODIPY FL is conjugated to the phosphate of the 5’-terminal cytosine of one RNA strand. The labeled BODIPY-RNA strand is annealed to a nucleic acid in which a complementary guanosine residue is base paired directly opposite the fluorescently labeled cytosine. The guanine nucleobase serves as a natural quencher of BODIPY FL [14–16]. Cleavage of fluorogenic duplex substrates of DICER and AGO2 produces short, unstable duplexes that dissociate at assay temperature (37°C) resulting in increased fluorescence intensity (Fig. 1). Sequences of individual strands are shown in Table 1. Names of ssRNA sense and antisense strands are denoted with the suffix “S” or “AS,” respectively. Names of fluorogenic duplex substrates lack the suffix. Fluorogenic substrates and cleavage products can be analyzed by thermal denaturation and annealing without adding exogenous intercalating fluorescent dyes.

### Duplexing and quenching analysis of fluorogenic dsRNA substrates

**Fig 2:**
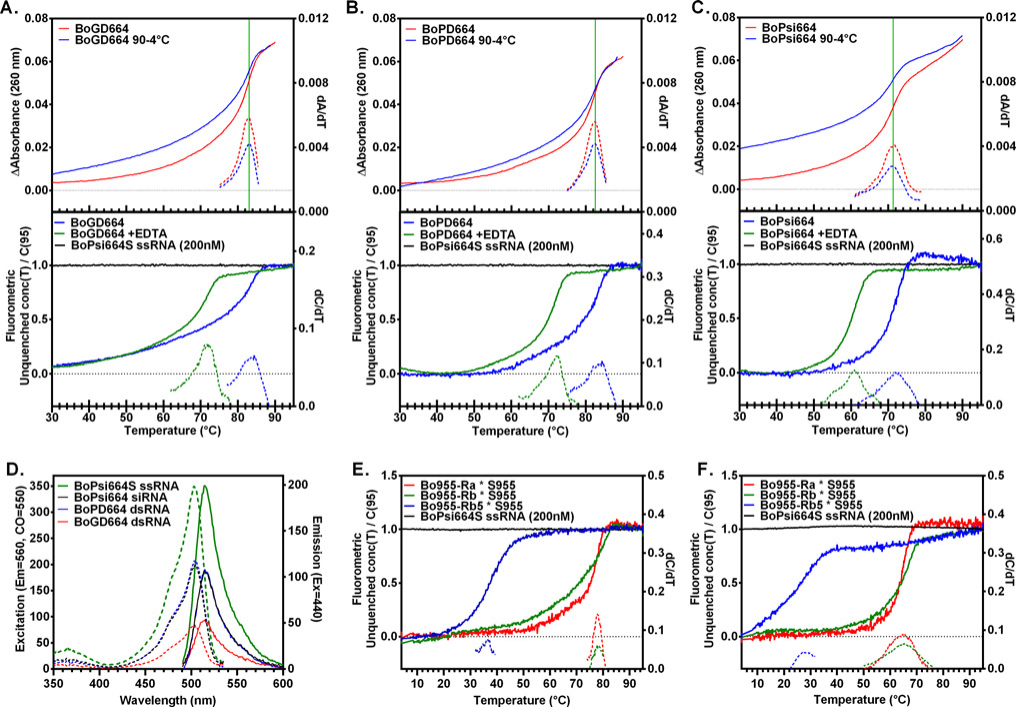
Duplex stability during dsRNA annealing and melting was measured using UV and fluorimetric methods. Single-stranded RNA conjugated to fluorescent BODIPY FL dye and quencherless ssRNA (200 nM each strand) was slowly annealed (upper panels of **A, B, C**; blue) or melted (upper panels of **A, B, C**; red) as measured by UV-visible spectrophotometer with Peltier temperature controller (Absorbance, 260 nm). Annealing was measured fluorimetrically using an ABI 7900HT Real Time PCR System (lower panels of **A, B, C**) with the temperature-dependent concentration of unquenched strand normalized to unquenched strand concentration at T = 95°C. Duplexes BoGD664 (**A**), BoPD664 (**B**), BoPsi664 (**C**), Bo955-Ra, Rb and Rb5 duplexed to ssRNA S955 (**E, F**) were analyzed in Assay Buffer (**A-E**) or Assay Buffer containing 5 mM EDTA (**F**). Fluorescence excitation spectra (Em = 560 nm, dashed curves) and emission spectra (Ex = 440 nm, solid curves) show quenching of dsRNA (BoPsi664, BoPD664 or BoGD664) vs. the control unquenched ssRNA BoPsi664S with no shift in excitation or emission peaks (**D**). First derivatives are displayed using dashed lines (**A-C, E-F**).

We analyzed RNAi substrates that target human *HIF1A* including the DICER substrate BoGD664, BoPD664 and fluorogenic siRNA BoPsi664 to determine whether the stability of BODIPY FL-labeled dsRNA duplexes can be measured fluorimetrically. By slowly increasing or decreasing temperature, we measured the melting and annealing of BODIPY FL-dsRNA duplexes by conventional UV spectrophotometry (Absorbance at 260 nm), and melting temperatures (*T*_m_) were measured at the peak of first derivative (dA_260_/dT). Thus, DICER substrate BoGD664, BoPD664 and fluorogenic siRNA BoPsi664 had UV *T*_m_ of 83.0, 82.5 and 71.3°C, respectively (Fig. 2A-C, upper panels). During slow annealing, fluorimetric measurements of unquenched strand concentration as a function of temperature C(*T*) and total unquenched strand concentration measured at *T* = 95°C, C(95) were recorded using an 7900HT Real Time PCR System (Applied Biosystems Inc., Foster City, CA). The fraction of unquenched strands was given by C(*T*)/C(95), and the fluorimetric *T*_m_ was measured at the peak of the first derivative with respect to temperature. The UV melting temperatures for BoGD664, BoPD664 and BoPsi664 (Fig. 2A-C, upper panels) were consistent with the fluorimetric *T*_m_ of 83.4, 83.4 and 72.2°C, respectively (Fig. 2A-C, lower panels). The effect of divalent cations on duplex stability was tested by addition of EDTA, which decreased the fluorimetric melting temperatures of BoGD664, BoPD664 and BoPsi664 (Δ*T*_m_ = -11.4, -11.2 and -11.4°C, respectively).

Next, quenching of BODIPY FL-RNA was tested for any effect on fluorescence excitation, emission and quenching efficiency. Spectral scans of unquenched BODIPY FL-labeled ssRNA (BoPsi664S) revealed the fluorescence excitation peak at 504 nm and an emission peak at 515 nm (Fig. 2D). Quenched dsRNAs including the siRNA duplex BoPsi664 and duplex DICER substrates (BoPD664 and BoGD664) share the excitation and emission peaks of the ssRNA BoPsi664S (Fig. 2D). Efficient quenching was observed for BoGD664 (labeled guide strand of blunt end dsRNA) at the excitation peak (Q = 0.76) and emission peak (Q = 0.73). Other dsRNAs had fluorescent label at the opposite end of the duplex. BoPD664 and BoPsi664 (labeled passenger strand annealed to strand with 3’-dTdT dinucleotide overhang) had quenching efficiencies of Q = 0.41 and 0.43 at the excitation peak and Q = 0.46 and 0.47 at the emission peak. Quenching at a blunt end is more efficient than quenching opposite a strand with an overhang.

To show generality, we examined fluorogenic dsRNA substrates targeting a different sequence (human *TYMS* gene encoding thymidylate synthase). BODIPY FL-labeled ssRNA (Bo955-Ra (24 nt), Bo955-Rb (31 nt) or Bo955-Rb5 (10 nt)) was slowly annealed to synthetic *TYMS* RNA, S955 (50 nt) in Table 1. In the presence of 1 mM free Mg^2+^, annealing of Bo955-Ra*S955, Bo955-Rb*S955 and Bo955-Rb5*S955 show fluorimetric *T*_m_ of 77.9, 78.3 and 36.3°C, respectively (Fig. 2E). In the presence of EDTA, annealing of Bo955-Ra*S955, Bo955-Rb*S955 and Bo955-Rb5*S955 show fluorimetric *T*_m_ of 64.7, 65.0 and 27.7°C, respectively (Fig. 2F). Chelation of divalent cations also shifted melting temperatures of the *TYMS* duplex series; Bo955-Ra*S955, Bo955-Rb*S955 and Bo955-Rb5*S955 show fluorimetric Δ*T*_m_ of-13.2, -13.3 and-8.6°C, respectively. *TYMS*-targeting fluorogenic dsRNA substrates shared biophysical characteristics with the series of *HIF1A*-targeting of RNAi substrates including thermal stability of dsRNA duplexes that increases with dsRNA length and a distinct contribution of divalent cations to dsRNA stability.

### Protein expression and purification

**Fig 3:**
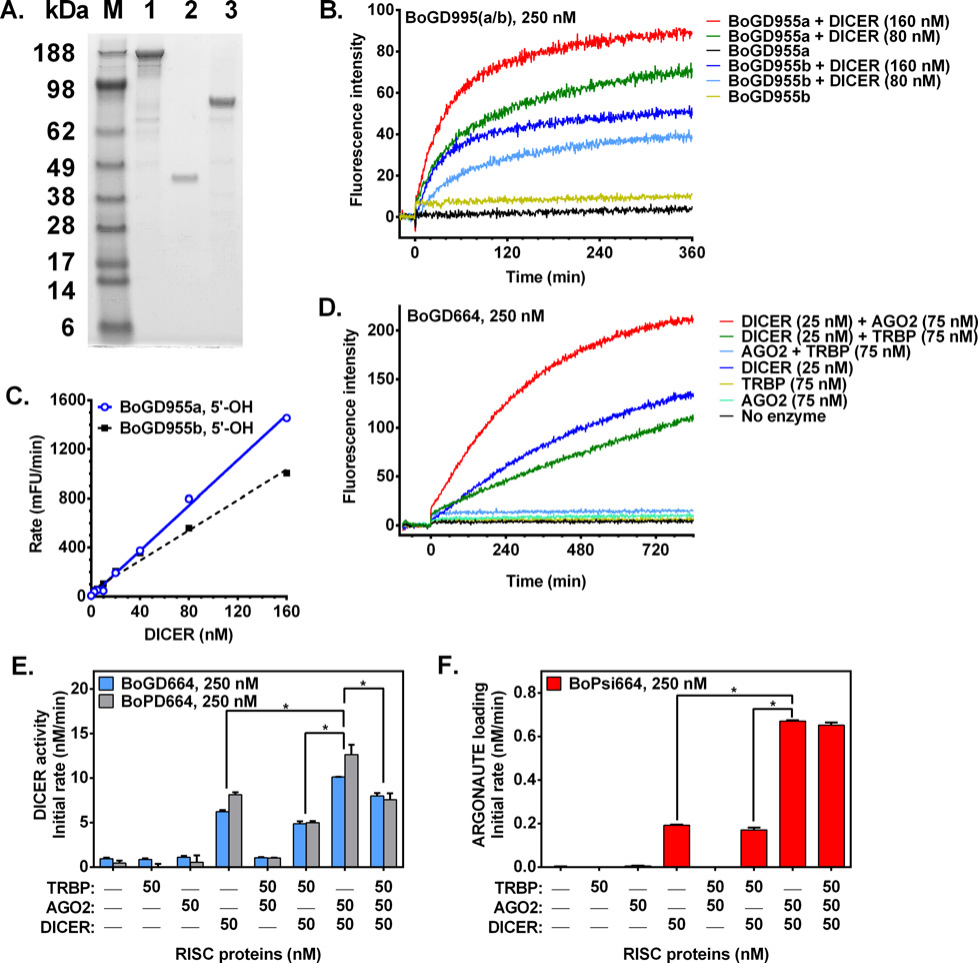
“Quencherless” fluorogenic substrates allow real-time monitoring of enzymatic activity of DICER and reconstituted RISC complex for enzyme kinetics assays. Recombinant human RNAi proteins were expressed in insect cells, purified and analyzed by SDS-PAGE (2.5 μg/lane) with Coomassie Blue staining (**A**). Lanes: SeeBlue Plus2 pre-stained markers (M), DICER (1), TRBP (2) and AGO2 (3). Continuous enzymatic assays of DICER using fluorogenic substrates (250 nM) bearing asymmetric overhangs (3’-dinucleotide overhang on anti-sense strand and long sense overhang) are shown (**B**), and linearity with enzyme concentration is shown (**C**). Continuous assays of reconstituted human RISC (combinations of purified enzymes) using DICER substrate BoGD664 (250 nM; **D-E**) or fluorogenic siRNA (AGO-loading substrate BoPsi664; 250 nM; **F**). AGO2 increases enzymatic activity of reconstituted RISC: (DICER+AGO2 > DICER alone (**E-F**). AGO2 or TRBP alone show no activity (**E-F**). *, p<0.05.

To study enzyme mechanisms in RNA interference, three components of the RISC complex (recombinant human DICER1, AGO2 and the TARBP2 variant of TRBP were expressed in insect cells and purified with metal affinity and size-exclusion chromatography. SDS-PAGE analysis (Fig. 3A) shows the purified proteins AGO2, TRBP and DICER had an apparent molecular weight of 87 kDa, 42 kDa and ~188 kDa, respectively. By mass spectrometry, AGO2 had a molecular mass of 97,119 compared to the theoretical value of 97,133 for the construct. Purified TRBP was also confirmed as full length by MS analysis.

### Fluorogenic DICER substrates targeting human thymidylate synthase (*TYMS*)

DICER activity was monitored using fluorogenic DICER substrates with asymmetric overhangs (3’-dinucleotide overhang on anti-sense strand and a 21–28-nt overhang on the sense strand. After cleavage of BoGD955a and BoGD955b, the labeled duplex products dissociate at the 37°C assay temperature as indicated by increase in fluorescence intensity (Fig. 3B). Enzymatic activity (measured by initial rates) was linear with concentration of DICER enzyme (Fig. 3C). Although the enzymatic reactions contained two-fold differences in enzyme concentration, as reactions proceeded toward the endpoint, fluorescence intensities approached the same high level, which is consistent with complete enzymatic conversion of substrate to products. In the event of enzyme-substrate binding without catalysis, the observed endpoint fluorescent intensities would have differed depending upon DICER concentration as in a binding assay, but this was not observed. Since the fluorogenic assay of *TYMS*-targeting substrates was consistent with DICER enzymatic activity, we expanded the fluorogenic assay to unrelated sequences that target the *HIF1A* gene.

### Fluorogenic DICER substrates and siRNA targeting human *HIF1A*

A series of duplex substrates with a different 19-mer core sequence was designed to serve as substrates of purified RNAi enzymes (Fig. 1). Fluorogenic DICER substrates were labeled on Guide Strand (BoGD664) or Passenger Strand (BoPD664), and a fluorogenic siRNA labeled on the Passenger Strand (BoPsi664) was prepared (Table 1). Fluorescence intensity was monitored for enzymatic cleavage of DICER substrates and siRNA by combinations of the purified RNAi enzymes DICER, AGO2 and the dsRNA-binding protein TRBP, and monitoring of BoGD664 cleavage is shown (Fig. 3D). As expected neither AGO2, TRBP, nor the combination AGO2+TRBP exhibited any activity upon DICER substrate as measured by initial rates. Purified DICER demonstrated activity with both the DICER substrates labeled on the 5’ end of either strand (Fig. 3E). For cleavage of DICER substrates, AGO2 was inactive, but the combination of DICER+AGO2 had higher activity than DICER alone (Fig. 3E) suggesting that the DICER-AGO2 enzyme complex (a subset of the RISC complex) exhibits functional interactions that could enhance the processing of DICER substrates. Surprisingly, addition of a third member of the RISC complex (dsRNA-binding protein TRBP) decreased the apparent fluorogenic activity of DICER+AGO2 (Fig. 3E). Thus, the DICER-AGO2 enzyme complex enhances the processing of DICER substrates.

Since siRNA is an intermediate in the RISC pathway, we also tested the fluorogenic siRNA for processing by RISC components. In AGO2 loading, the product of DICER cleavage (siRNA) binds to the active site of AGO2 where the Passenger Strand is cleaved and dissociates leaving the Guide Strand loaded on AGO2. In the AGO2 loading assay, AGO2, TRBP and AGO2+TRBP did not exhibit any activity upon fluorogenic siRNA, and DICER showed little activity (Fig. 3F). The combination of DICER+AGO2 enzymes had higher activity than DICER alone for cleaving the fluorogenic siRNA, and activity was unaffected by TRBP (Fig. 3F). Taken together, these data suggest functional interactions of the DICER-AGO2 enzyme complex that both enhance the cleavage of DICER substrates and that enhance processing of the siRNA intermediate by AGO2.

### Functional interaction of AGO2 and DICER enzymes

The reconstituted RISC complex (combination of AGO2 + DICER enzymes) demonstrated a synergistic increase in enzymatic activity with both DICER substrates (Fig. 3E) and with fluorogenic siRNA (Fig. 3F). Enzyme combinations with TRBP did not enhance the apparent enzymatic activity (Fig. 3E-F).

**Fig 4:**
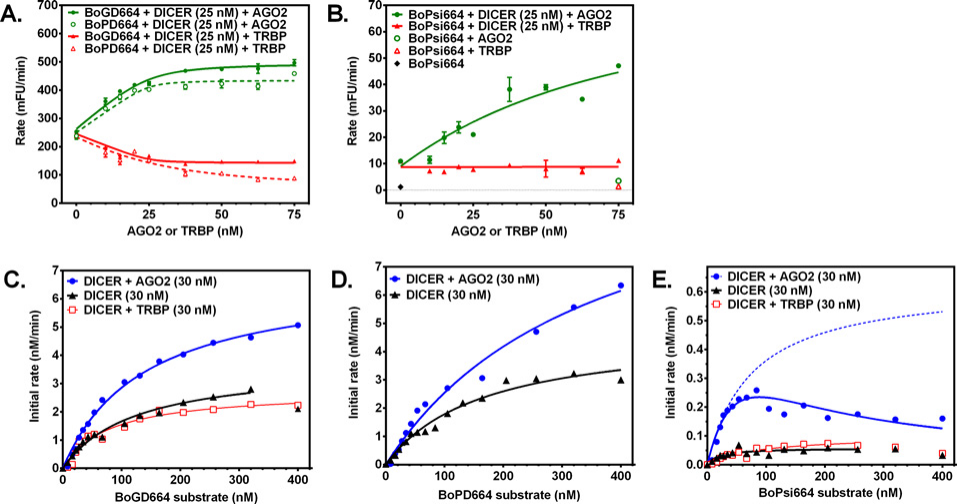
Binding interactions in the RISC complex are functionally determined using enzyme kinetics. DICER-AGO2 binding interaction was assessed by enzymatic activity assays modeled using the Morrison equation [17]. Titration of DICER (25 nM) with AGO2 enhances enzymatic activity for cleavage of either fluorogenic DICER substrate (250 nM; **A**) and the fluorogenic siRNA (AGO-loading substrate; 250 nM; **B**) apparently via high-affinity binding interaction. AGO loading is dependent on AGO2 concentration and requires DICER (**B**). Michaelis-Menten kinetics were observed for DICER and minimal reconstituted RISC using both DICER substrates (**C-D**). AGO-loading siRNA exhibited kinetics consistent with a substrate inhibition model (**E**), whereas DICER or DICER+TRBP demonstrate minimal enzymatic activity. Lesser apparent fluorogenic activity was observed in combination with the dsRNA-binding protein TRBP.

Interactions in the RISC complex were assessed by titrating DICER enzyme with AGO2 or TRBP and measuring activity. DICER substrates labeled on the 5’ end of either strand were cleaved by DICER in an AGO2-dependent manner (Fig. 4A). By contrast, titration of DICER with TRBP showed a small concentration-dependent decrease in apparent activity (Fig. 4A). No further increase in enzymatic activity was observed after exceeding a 1:1 molar ratio of AGO2: DICER (Fig. 4A). Fitting the enzymatic activity curves (Fig. 4A) using the Morrison equation [17] resulted in low nanomolar dissociation constants, suggesting a functional high-affinity interaction between AGO2 and DICER enzymes in the reconstituted RISC complex (*K*_d,app_ = 2.2 and 0.54 nM using DICER substrates BoGD664 and BoPD664, respectively). The DICER + AGO2 enzyme combination cleaved the fluorogenic siRNA in an AGO2-dependent manner, whereas TRBP had no effect on apparent activity (Fig. 4B). These results suggest that DICER binds AGO2 in an equimolar ratio and that the DICER-AGO2 enzyme complex demonstrates functional interactions between these enzymes that enhance the processing of DICER substrates and siRNA intermediates.

### Enzyme kinetics

Next, mechanisms that underlie functional interactions of enzyme components of the RISC complex were investigated using substrates and an intermediate utilized in the enzyme complex. We characterized enzyme kinetics for DICER cleavage and AGO2 loading. Initial rate data were collected for DICER alone and for combinations of DICER with AGO2 or TRBP (Fig. 4C-E), and kinetic parameters were listed in Table 2. Using either DICER substrate, normal Michaelis-Menten kinetics were observed for DICER and DICER+AGO2 enzymes (Table 2). DICER enzyme cleaved both DICER substrates readily; selectivity was similar for BoGD664 (*k*_cat_/*K*_m_ = 2360 M^-1^ s^-1^) and BoPD664 (*k*_cat_/*K*_m_ = 1890 M^-1^ s^-1^) in Table 2. However, the DICER+AGO2 complex did not process both DICER substrates equally. DICER+AGO2 favored processing of BoGD664 substrate (relative to BoPD664) as evidenced by improved selectivity (1.8-fold *k*_cat_/*K*_m_) and 2.6-fold improved apparent *K*_m_ (Table 2).

**Table 2:** Kinetic parameters for reconstituted human RISC (37°C).

dsRNA substrate	Purified recombinant RISC proteins	*K*_m,app_ (nM)	*k*_cat_ (s^-1^)	Substrate Inhibition, *K*_i_ (nM)	*k*_cat_ / *K*_m,app_ (M^-1^ s^-1^)
BoGD664	DICER	122	2.88×10^-4^	-	2360
	DICER+AGO2	139	5.31×10^-4^	-	3820
	DICER+TRBP	77	2.14×10^-4^	-	2770
BoPD664	DICER	165	3.11×10^-4^	-	1890
	DICER+AGO2	359	7.68×10^-4^	-	2140
BoPsi664	DICER	19	6.69×10^-6^	-	344
	DICER+AGO2	74	7.28×10^-5^	105	979
	DICER+TRBP	85	1.16×10^-5^	-	137

### Kinetic evidence for binding interactions in reconstituted RISC complex

During processing of dsRNAs in the DICER-AGO2 complex, siRNA is an intermediate (product of DICER and substrate of AGO2). Because mechanistic detail for siRNA processing in the DICER-AGO2 complex is unknown, it was of interest to determine experimentally the mechanism by feeding a fluorogenic intermediate (siRNA) into the DICER-AGO2 enzyme complex to determine how it is processed. Reconstituted RISC (AGO2+DICER) was supplied increasing concentrations of the fluorogenic siRNA BoPsi664. At low concentrations of fluorogenic siRNA, increasing initial rates were observed consistent with Michaelis-Menten kinetics (Fig. 4E, dotted line). However, as concentrations of fluorogenic siRNA were further increased, the observed initial rates began to decline (Fig. 4E, solid blue line) and departed from normal Michaelis-Menten kinetics (Fig. 4E, dotted line). Thus, the observed enzyme kinetics for DICER+AGO2 processing of BoPsi664 were consistent with substrate inhibition for the BoPsi664 (Fig. 4E) with *K*_i_ (105 nM) at 1.4 times the apparent *K*_m_ of 74 nM (Table 2). Substrate inhibition observed using the DICER+AGO2 complex suggests that fluorogenic siRNA binds two classes of sites in the DICER+AGO2 complex. Occupation of one site by siRNA could inhibit processing at the other site in the enzyme complex. For example, one fluorogenic siRNA molecule bound to the active site of DICER and a second fluorogenic siRNA bound to the active site of AGO2 may be in close proximity. At high concentrations of siRNA, active sites of both enzymes are occupied, and the direct transfer of DICER-bound siRNA (product) to AGO2 is inhibited by another siRNA molecule already bound as a substrate in the active site of AGO2. The substrate inhibition of DICER-AGO2 complex by fluorogenic siRNA is consistent with a direct transfer mechanism in which the product of DICER (siRNA) is directly transferred to AGO2 in the enzyme complex.

### Substrate specificity

**Fig 5:**
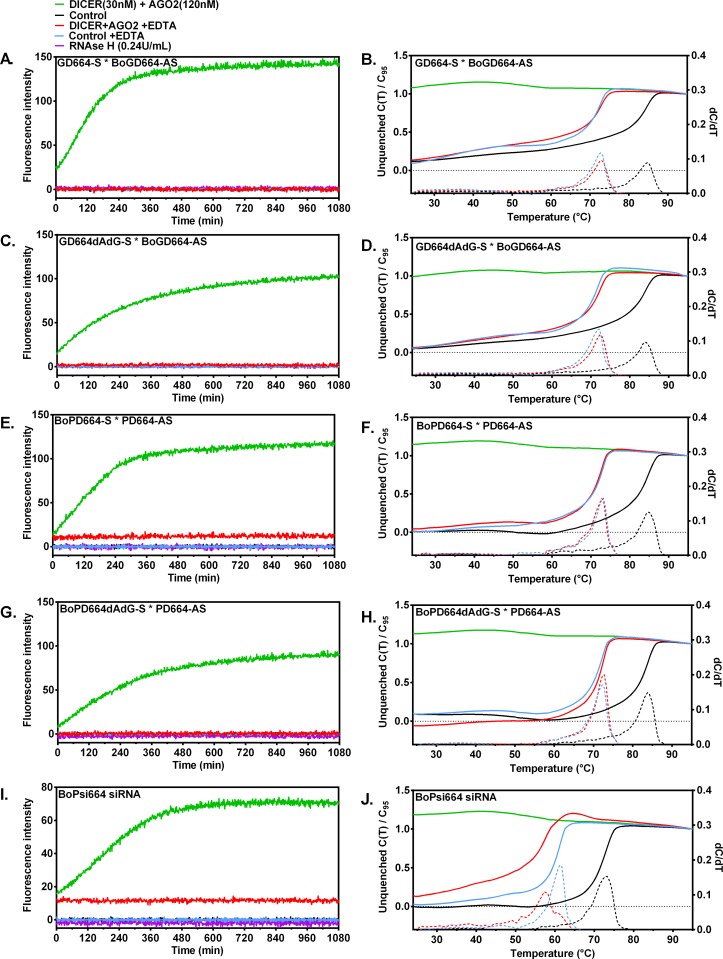
DICER+AGO2 enzymes cleave fluorogenic substrates into duplex products that are unstable at T>25°C. Enzymatic activity (37°C) is shown for the dsRNA DICER substrates (BoGD664AS * GD664S, **A**; BoGD664AS-dAdG * GD664S, **C**; BoPD664S * PD664AS, **E**; BoPD664S-dAdG * PD664AS, **G**) and fluorogenic siRNA BoPsi664 (BoPsi664S * Psi664AS; **I**) by AGO2 (120 nM) + DICER (30 nM) but not by *E*. *coli* RNase H (0.24 U/mL). Melting analysis shows that only products of DICER+AGO2 cleavage are already melted at *T* ≥ 25°C, whereas control reactions containing either no enzyme or RNAse H instead of DICER+AGO2 have fluorimetric dsRNA stability (as measured by *T*_m_) that is indistinguishable from *T*_m_ of the substrates (**B, D, F, H, J**). EDTA inhibits enzymatic cleavage of substrates and gives the characteristic decrease in *T*_m_ compared to Assay Buffer containing divalent cations (**B, D, F, H, J**). First derivatives are displayed using dashed lines.

DICER substrates typically bind DICER enzyme via three key molecular interactions: (1.) the 3’-dinucleotide overhang binds the PIWI/Argonaute/Zwille (PAZ) domain of DICER, (2.) dsRNA helix forms electrostatic interactions with the basic binding trench of DICER, and (3.) in DICER’s active site the dsRNA helix undergoes double-stranded endonucleolytic cleavage leaving the 5’-terminal nucleotide of the Guide Strand bound by the MID domain of DICER. However, DICER’s unexpected cleavage of DICER substrate BoPD664 (with increased fluorescence in Fig. 3E, 4D) could be the result of the blunt end binding near the PAZ domain of DICER resulting in endonucleolytic cleavage near the fluorescent label followed by increased fluorescence signal. DICER substrates that are designed with terminal deoxy dinucleotides on the blunt 3’ terminus of the passenger strand are reported to be preferentially cleaved by DICER in the intended orientation [18] resulting in improved potency and efficacy in cultured cells [19]. Therefore, Passenger Strands that previously contained terminal ribo AG nucleotides (BoGD664 and BoPD664) were synthesized with terminal deoxy dinucleotides (dAdG) and annealed to form the blunt-ended duplexes with RNA. The 3’-dTdT overhang was retained on the Guide Strand (BoGD664dAdG and BoPD664dAdG). Fluorogenic DICER substrates (200 nM) either with (Fig. 5C-D, G-H) and without (Fig. 5A-B, E-F) the deoxy dinucleotide modification in the Passenger Strand [18] and the fluorogenic siRNA BoPsi664 (Fig. 5I-J) were assayed for cleavage by reconstituted RISC (DICER+AGO2) and by *Escherichia coli* ribonuclease H. No enzymatic activity was observed using reconstituted RISC in the presence of EDTA or using RNase H. DICER+AGO2 cleaved the Guide-Strand labeled DICER substrates (200 nM) at an initial rate ratio of 0.63 (BoGD664dAdG: BoGD664). Similarly, the ratio of DICER+AGO2 activity for Passenger-Strand labeled substrates (BoPD664dAdG: BoPD664) was 0.58. These results suggest the DICER substrate design BoPD664 (without the Passenger Strand modification) may have been cleaved in the unintended orientation 42% of the time and support the importance of the Passenger Strand modification.

### DICER+AGO2 enzymes cleave fluorogenic substrates into duplex products that are unstable above 25°C

The products of the enzymatic assays were analyzed by thermal denaturation using an iCycler iQ5 Real Time system (Bio-Rad Laboratories, Hercules, CA). Substrates without treatment by enzymes exhibit distinct melting transitions (Fig. 5, black curves) characteristic of the dsRNA duplex stability. Control conditions in which magnesium ion (critical for catalysis) is chelated by EDTA show that substrates treated with enzyme do not result in detectable consumption of substrate, and the *T*_m_ values are 11.5 to 12.0°C lower than conditions containing 1 mM free Mg^++^ (Fig. 5B, D, F, H, J; Table 3). However, complete reaction conditions including substrate, RISC enzymes (AGO2+RISC) and 1 mM free Mg^++^ resulted in complete consumption of substrate as evidenced by loss of dsRNA melting transition (melting not detectable at *T*≥25°C; Fig. 5B, D, F, H, J; DICER+AGO2). By contrast for control conditions, melting was observed that was indistinguishable from dsRNA substrate alone (Fig. 5B, D, F, H, J; Control). These results demonstrate that magnesium-dependent catalysis by DICER+AGO2 enzymes (but not RNase H control) cleaves the dsRNA DICER substrates and fluorogenic siRNA to yield products that cannot persist as duplexes at assay temperature (37°C). Following cleavage by DICER and AGO2, the shortened products are unstable duplexes that dissociate, and fluorescence intensity increases.

**Table 3:** RNA duplex stability (*T*_m_) measured by fluorescence quenching of BODIPY FL-labeled RNA.

Duplex	Observed *T*_m_ (°C)		Calculated *T*_m_ (°C)	
	Substrate + Mg^++^ (1 mM)	Substrate, Mg-free (EDTA)	Substrate + Mg^++^ (1 mM)	DICER+AGO2 Product + Mg^++^ (1 mM)
BoGD664 (200 nM)	84.7	72.7	77.9	15.0
BoGD664dAdG (200 nM)*	84.0	72.0	77.1	<10
BoPD664 (200 nM)	84.8	73.0	77.9	24.7
BoPD664dAdG (200 nM) *	83.8	72.3	77.1	24.7
Psi664 (80 nM)	73.0	61.3	64.3	24.7

* Right (“R”) DICER substrate with two deoxyribonucleotides on the 5’ terminus of sense strand [18].

### Competitive substrate assays

**Fig 6:**
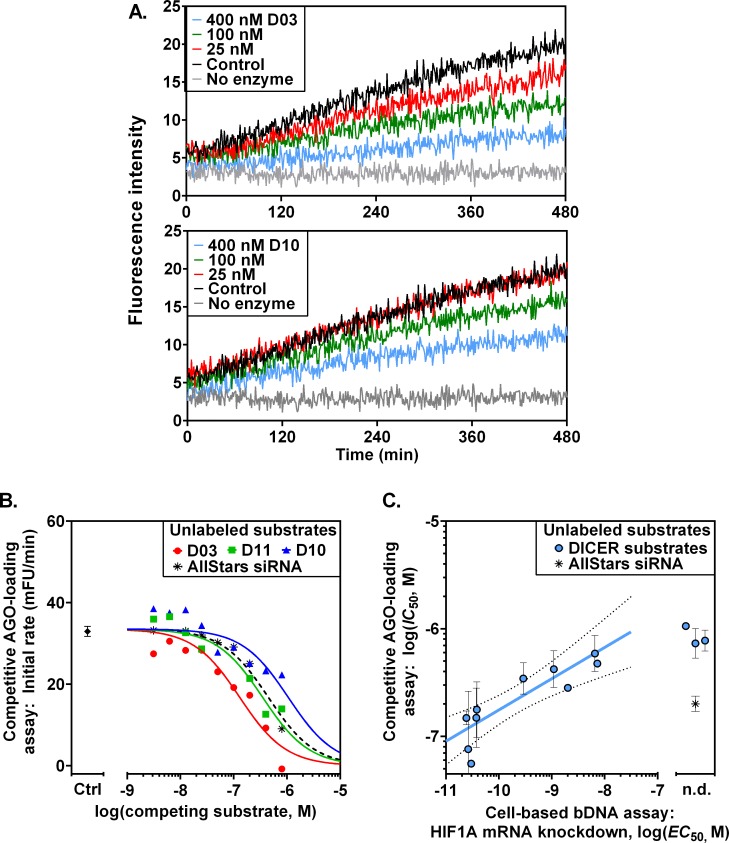
Competitive ARGONAUTE2 loading in the DICER-AGO2 enzyme complex correlates with efficacy in cell-based RNAi assays. Kinetic assays of enzymatic AGO loading were performed by applying DICER+AGO2 (30 nM each) to mixtures containing a fixed concentration of fluorogenic AGO substrate (siRNA BoPsi664, 80 nM) and variable concentrations (0, 25, 100, 400 nM shown) of unlabeled competing DICER substrate D03 (**A**, panel above) or DICER substrate D10 (**A**, panel below). “No enzyme” control wells contain BoPsi664 without DICER or AGO2. The apparent initial rate of fluorogenic AGO loading was dependent upon the concentration of unlabeled competing DICER substrates D03, D10 and D11 (**B**, colored symbols) or unlabeled competing AGO substrate (**B**, AllStars siRNA) compared to control (**B**, “Ctrl”). The potency of competitive AGO loading of DICER substrates by the DICER-AGO2 complex (*IC*_50_) was correlated to *HIF1A* mRNA in Huh-7.5 cells as detected by branched DNA (bDNA) assay (**C**). Dotted lines indicate the 90% confidence interval, and error bars show SEM. “n.d.,” not detectable.

In order to rank the ability of unlabeled RNAi substrates to be processed by enzymes of the RISC complex, a competitive substrate assay was developed. In competitive AGO2-loading assays, a fixed concentration of fluorogenic siRNA substrate was combined with increasing concentrations of unlabeled DICER substrates or unlabeled siRNA. The DICER-AGO2 complex was added, and reaction progress was monitored to measure the apparent (fluorogenic) initial rates of reaction. The enzymatic rates for reconstituted RISC exhibited competition was dependent on the concentrations of competing unlabeled DICER substrate (D03) as low as 25 nM (Fig. 6A, upper panel) compared to control without unlabeled substrate (Fig. 6A, Control). By contrast, higher concentrations (≥100 nM) of the unlabeled DICER substrate D10 were required to affect the initial rate of AGO loading. The apparent fluorogenic initial rates for AGO loading were found to decrease with increasing concentrations of unlabeled DICER substrates (D03, D11, D10) or unlabeled AllStars siRNA compared to the control without unlabeled substrate (Fig. 6B, diamond). This result is consistent with alternative substrate (unlabeled siRNA or unlabeled DICER substrate) competing with fluorogenic substrate for the AGO2 enzyme active site. The rank order of potency of unlabeled DICER substrates in the competitive AGO2-loading assay was D03 > D11 > AllStars siRNA > D10.

### Correlation of enzymatic assay for competitive AGO2 loading *in vitro* vs. cell-based RNA interference assay

To evaluate biological relevance, the enzymatic assay of competitive AGO2 loading of unlabeled DICER substrates by the DICER•AGO2 complex was tested vs. a cell-based assay of *HIF1A* mRNA knockdown. A series of DICER substrates was tested for knockdown of *HIF1A* mRNA in Huh-7.5 cells. For bDNA detection of HIF1A knockdown reveals an observed rank order of potency (*IC*_50_) for the DICER substrates (D03 > D11 > D10), which was consistent with rank order in the competitive enzyme assay for AGO2 loading in reconstituted RISC (Table 4). The control (non-silencing AllStars siRNA) did not knock down *HIF1A* mRNA in the cell assay. As expected, however, AllStars siRNA competed with the fluorogenic siRNA for AGO2 loading in the enzyme assay (*IC*_50_ = 209±69 nM; Table 4). Competitive AGO loading (enzymatic *IC*_50_ *in vitro*) of unlabeled DICER substrates by the DICER•AGO2 complex was correlated with potency (*EC*_50_) in the cell-based assay of *HIF1A* mRNA knockdown (Fig. 6C). The DICER substrate series shows a log-log correlation slope of 0.29. Thus among members of the DICER substrate series, a 10-fold improvement in the relative ability of substrate to be processed and loaded onto AGO2 has a strong (~2800-fold) effect upon potency for mRNA knockdown, which may reflect catalytic degradation of multiple mRNA copies by each AGO2-Guide Strand complex. The enzyme kinetics assay suggests that certain sequence-specific aspects of the tested designs of DICER substrates were more compatible for processing by the DICER•AGO2 complex for loading AGO2 with the appropriate Guide Strand, and these results were correlated with knockdown in the cell assay.

**Table 4:** Competitive AGO2-loading enzymatic assay *in vitro* vs. knockdown of HIF1A mRNA in Huh 7.5 cells (bDNA assay).

Competing substrate	dsRNA sequence	HIF1A mRNA knockdown (bDNA assay) *EC*_50_ (nM)	AGO2-loading by competitive enzymatic assay *IC*_50_ (nM)
D03	5'- CCGGUUGAAUCUUCAGAUAUGAAAA-3' 3'-UUGGCCAACUUAGAAGUCUAUACUUUU-5'	0.024	150
D02	5'- GCUGAUUUGUGAACCCAUUCCUCAC-3' 3'-CACGACUAAACACUUGGGUAAGGAGUG-5'	0.026	142
D01	5'- ACCGGUUGAAUCUUCAGAUAUGAAA-3' 3'-UUUGGCCAACUUAGAAGUCUAUACUUU-5'	0.030	56
D04	5'- GUUCCUGAGGAAGAACUAAAUCCAA-3' 3'-GUCAAGGACUCCUUCUUGAUUUAGGUU-5'	0.038	180
D05	5'- GGAACAUGAUGGUUCACUUUUUCAA-3' 3'-UACCUUGUACUACCAAGUGAAAAAGUU-5'	0.038	209
D11	5'-ACCUGAGCCUAAUAGUCCCAGUGAAUA-3' 3'-UGGACUCGGAUUAUCAGGGUCACUU-5'	0.29	364
D12	5'-UACUCAGGACACAGAUUUAGACUUGGA-3' 3'-AUGAGUCCUGUGUCUAAAUCUGAAC-5'	1.1	453
D07	5'- GGAUAAAAUUCUCAAUUCAGAGAAA-3' 3'-AACCUAUUUUAAGAGUUAAGUCUCUUU-5'	2.0	282
D10	5'-CCUGAGGAAGAACUAAAUCCAAAGAUA-3' 3'-GGACUCCUUCUUGAUUUAGGUUUCU-5'	6.5	633
D06	5'- GGAUAGUGAUAUGGUCAAUGAAUUC-3' 3'-CACCUAUCACUAUACCAGUUACUUAAG-5'	7.2	474
D09	5'-CAGACACCUAGUCCUUCCGAUGGAAGC-3' 3'-GUCUGUGGAUCAGGAAGGCUACCUU-5'	n.d.	767
D25	5'-GCACCCCCACCUCUGGACUUGCCUUUC-3' 3'-CGUGGGGGUGGAGACCUGAACGGAA-5'	n.d.	796
D08	5'-CCAGAAGAACUUUUAGGCCGCUCAAUU-3' 3'-GGUCUUCUUGAAAAUCCGGCGAGUU-5'	n.d.	1060
AllStars	Proprietary siRNA (Qiagen, Inc.)	n.d.	209

n.d.: Not detectable.

## Discussion

This study describes mechanistic details for RNA processing by enzymes of the human RISC complex. New mechanistic findings in RNAi were made possible by the development of continuous enzymatic assays that provided detailed enzyme kinetics data. Fluorogenic substrates were synthesized comprising a fluorescent strand (ssRNA covalently attached BODIPY FL fluorescent dye) annealed to a complementary ssRNA. Annealing of the BODIPY FL-labeled cytosine residue to guanosine of the complementary strand causes fluorescence quenching by the guanosine base. The resulting “quencherless” fluorogenic substrates allowed continuous enzymatic assays of DICER and AGO2 for enzyme kinetics studies. We studied fluorogenic substrates targeting two different human genes (*TYMS* and *HIF1A*) and determined that particular combinations of proteins of the RISC complex can process certain dsRNA structures. Purified DICER enzyme (but not AGO2) cleaves fluorogenic DICER substrates, and the combination DICER+AGO2 (enzyme components of the RISC complex) synergistically increases the enzymatic activity via a functional high-affinity interaction between DICER and AGO2 enzymes. Addition of a third RISC component, the dsRNA-binding protein TRBP slightly decreased the apparent fluorogenic activity. DICER and DICER+AGO2 activity upon DICER substrates exhibited Michaelis-Menten kinetics. These results suggest that interactions in the DICER+AGO2 complex are important for processing DICER substrates. We further studied the interaction using siRNA, an intermediate in the RISC pathway. The combination of DICER+AGO2 enzymes (but not AGO2) cleaved fluorogenic siRNA, and the addition of TRBP did not affect the apparent enzymatic activity. The finding of substrate inhibition for DICER+AGO2 processing of fluorogenic siRNA suggests that the siRNA binds two sites during processing, most likely the active sites of the two enzymes. These data are consistent with a direct transfer mechanism in the DICER+AGO2 enzyme complex in which the siRNA product bound at the active site of DICER is directly transferred to the active site of AGO2 in the enzyme complex. The fluorogenic siRNA substrate was also used in competitive substrate assays to evaluate processing of unlabeled dsRNA therapeutic molecules by RNAi enzymes. In the competitive AGO2-loading assay, unlabeled synthetic dsRNAs (DICER substrates) were processed by DICER+AGO2 in competition with fluorogenic siRNA substrate. Unlabeled DICER substrates caused a concentration-dependent decrease in fluorogenic initial rates with *in vitro IC*_50_ values that correlate with *HIF1A* mRNA knockdown in Huh-7.5 cells. This result suggests that certain DICER substrate sequences had decreased efficacy in RNAi due to their poor ability to be processing by enzymes of the RISC complex. Finally, fluorogenic DICER substrates and fluorogenic siRNA were cleaved by DICER+AGO2 enzymes in a magnesium-dependent manner, and cleavage products do not exist as duplexes at assay temperature. We present a new method for studying mechanistic detail of RNAi enzymes using continuous enzyme assays. These data support the importance of the DICER+AGO2 enzyme complex for processing dsRNA molecules in the RNAi pathway. This method can be used to experimentally determine structure-activity relationships for synthetic dsRNA molecules including chemical modifications in order to diagnose issues with DICER cleavage and AGO2 loading.

This is the first study to demonstrate continuous monitoring for processing of dsRNA substrates by both DICER and AGO2 enzymes, which catalyze sequential steps in the RNAi pathway. Enzyme kinetics analysis revealed new findings regarding mechanism in the RISC complex. Continuous assays using dsRNA substrates show that enzymatic activity is functionally dependent upon high-affinity interaction between DICER and AGO2 enzymes. Further, enzyme kinetics using fluorogenic substrates provide evidence for a model in which the siRNA product of DICER is directly transferred to the active site of AGO2 enzyme in the RISC complex. Finally, the new fluorogenic assays demonstrate that unlabeled therapeutic RNAi molecules (*e*.*g*. synthetic dsRNA molecules) can be tested *in vitro* for their ability to be processed by DICER enzyme followed by AGO2 enzyme (AGO-loading activity), which correlates with mRNA knockdown activity in a cell-based RNAi assay.

Loading of AGO2 appears to occur by two pathways (*de novo* and reloading pathways). In the *de novo* pathway, DICER produces an siRNA molecule. The siRNA is transferred from the active site of DICER to its binding partner in the RISC complex, AGO2. Functionally in the RISC complex, AGO2 assumes a conformation that is competent for dsRNA binding and for cleavage. Passenger strand cleavage would result in AGO2 activation by programming AGO2 with the Guide Strand bound at the active site. Complexes of enzymes that catalyze sequential steps in an enzymatic pathway can improve flux through that pathway via direct transfer of the product of the first enzyme to the active site of the second enzyme. It would be most efficient for AGO2 activation if the siRNA product in DICER’s active site is transferred directly to AGO2 instead of being diluted into a cytoplasmic pool of ssRNA that is subject to ribonuclease degradation before it can accumulate to nanomolar concentrations (*K*_d_ ≥ 61 nM reported in reference [20] for loading onto AGO2). In the current study, substrate inhibition of AGO2-DICER complex using the fluorogenic siRNA could be explained by substrate binding to both AGO2 and DICER. The substrate inhibition observed in our study is consistent with the EM model for direct transfer in which opposite ends of an siRNA are each bound to the respective PAZ domains of the AGO2-DICER complex [21].

The second pathway for AGO2 loading is the reloading pathway, which would occur after the Guide Strand dissociates from the active site of AGO2. Guide-Strand loading onto AGO2 enzyme [20] in the absence of DICER would really be a reloading process that draws from a cytoplasmic pool of ssRNA Guide Strands. The released guide strands are very short and may lack structures such as the 5’-terminal 7-methylguanosine cap or 3’-polyadenylation that normally provides protection of mRNAs from exoribonucleases [22–24]. Therefore, guide strands of the cytoplasmic pool could be susceptible to degradation by intracellular ribonucleases, which would tend to decrease the importance of the reloading pathway. The AGO2-DICER complex mechanism could explain published observations that ssRNA can be loaded onto AGO2 and that dsRNA is not loaded due to poor binding by AGO2 alone [20], whereas dsRNA is loaded efficiently onto AGO2 in the AGO2-DICER complex.

“Quencher-less” fluorogenic assays offer benefits over current methodologies. RNAi is a complex, multi-step enzymatic pathway, and existing technologies typically do not allow individual activities in the pathway to be studied at large scale. Currently, assaying RNAi involves endpoint analysis at discrete time points that is expensive and/or labor-intensive usually involving radioactivity or PCR. Those methods provide a readout that must span the entire multi-step enzymatic pathway. By contrast, the current study can dissect the enzymatic pathway into smaller steps to determine where in the pathway that synthetic RNAi molecules function or fail to function. Novel continuous fluorogenic assays of ARGONAUTE loading and DICER enzymatic activity were developed to assess RNAi entities using reconstituted RISC from purified recombinant human enzymes. The new quencher-less method also minimizes perturbation of enzymatic activity by avoiding potential steric issues with bulky quencher molecules (typically large, fused aromatic systems) such as those required in a fluorogenic assay format for endonuclease assays of RNase H [25] and DICER [26]. Another report of fluorimetric detection of DICER activity relies on shifts in autocorrelation curves by fluorescence correlation spectroscopy [27]. One report has been published for fluorimetric detection of AGO2 Slicer activity [28], which is the enzymatic step immediately downstream of the DICER and AGO2-loading activities of the current report. Published applications that utilize quenching of BODIPY FL-labeled oligonucleotides are limited to PCR methods [14–16]. The quenching efficiencies of the four bases for many different fluorescent dyes including BODIPY FL have been tabulated, and of the four bases, guanosine was the best quencher [29,30]. Singlet-excited BODIPY FL is quenched by guanine through a photoinduced electron-transfer (PET) mechanism, which requires contact of excited fluorescent dye with quencher (van der Waals contact) for efficient quenching [29]. For DNA-BODIPY FL and dG in DNA duplexes, the charge separation that occurs during PET is fully reversible [29], which assures quantitative analysis in quenching studies.

Limitations of this study include a 3’-dTdT overhang in the fluorogenic substrates instead of the di-ribonucleotide overhang found in physiological DICER substrates. Since bases and ribose rings of the overhang nucleotides affect binding at the PAZ domain, the deoxynucleotide overhang could affect the enzyme kinetic parameters compared to physiological substrates of DICER and/or AGO2. Given that the physiological substrates of DICER and AGO can have many different sequences, the synthetic sequences of the fluorogenic substrates might also affect the enzyme kinetic parameters. However, those considerations do not negate the mechanistic findings for DICER and AGO2 in this study nor do they negate the utility of fluorogenic substrates for evaluation of the ability of synthetic RNAi molecules to compete with a given fluorogenic substrate molecule for processing by DICER+AGO2. Another limitation is that reconstituted RISC complex contains the dsRNA-binding protein TRBP in addition to DICER and AGO2 [4]. TRBP did not improve the apparent enzymatic activity of reconstituted RISC (DICER+AGO2) in this study; TRBP was dispensable as it is not required for dsRNA processing in the RISC complex *in vitro* [8,9].

## Conclusions

DICER and AGO2 enzymes form a functional equimolar complex with high affinity. This minimal RISC complex supports siRNA loading onto AGO2, whereas AGO2 does not efficiently self-load/cleave a double-stranded siRNA. Enzyme kinetics study of the minimal RISC supports a model in which the product of DICER is transferred directly to AGO2. “Quencherless” fluorogenic assays for enzymatic activity of DICER and AGO2-loading activity provide for continuous monitoring of RNAi enzymatic activity and enable detailed kinetic analysis on a scale that was previously not possible. These studies lay the foundation for a systematic biochemical approach to the DICER-AGO2 enzyme pathway for providing structure-activity relationships of duplex designs that correlate with mRNA knockdown in cell assays. Designs of RNAi molecules with low propensity to be loaded onto AGO2 can have limited effectiveness in RNAi-mediated knockdown. Conversely, an RNAi design with better efficiency for loading onto AGO2 can greatly amplify its effectiveness through the degradation of many mRNA molecules in multiple catalytic rounds of cleavage by AGO2 enzyme. The *in vitro* enzymatic assay of ARGONAUTE2 loading of DICER substrates was correlated with cell-based assay of mRNA knockdown, suggesting that improved DICER substrate designs with 10-fold greater processing by the DICER+AGO2 complex can provide a strong (~2800-fold) improvement in potency for mRNA knockdown. Optimizing the *in vitro* activity in designs of nucleic acid-based therapeutics is aimed at improving efficacy in the cell.
